# SEX HORMONES AND ARTHRITIS IN A LONG-LIVED ANIMAL MODEL, THE ELEPHANT

**DOI:** 10.1093/geroni/igz038.3370

**Published:** 2019-11-08

**Authors:** Daniella E Chusyd, Janine Brown, Tim R Nagy, Timothy Griffin, Virginia Kraus, Janet Huebner, David Allison, Steven N Austad

**Affiliations:** 1 Indiana University, Bloomington, Indiana, United States; 2 Smithsonian Conservation Biology Institute, Front Royal, Virginia, United States; 3 Department of Nutrition Sciences, University of Alabama at Birmingham, Birmingham, Alabama, United States; 4 Oklahoma Medical Research Foundation, Oklahoma City, Oklahoma, United States; 5 Duke University, Durham, North Carolina, United States; 7 Department of Biology, University of Alabama at Birmingham, Birmingham, Alabama, United States

## Abstract

Arthritis affects women more so than men, particularly after menopause. Sex hormones may play a role in arthritis development, yet this is still unclear. Elephants have a similar lifespan to humans and develop arthritis and joint issues (musculoskeletal impairments (MI)), but do not undergo menopause. Interestingly, many female zoo elephants do not exhibit normal ovarian cycles, closely resembling that in post-menopausal women. Objectives: 1) examine ovarian cyclicity status with MI (Asians: N=84; Africans: N=83); 2) determine whether gonadotropin and sex hormones are reduced in cycling elephants with MI (Asians: N=16; Africans: N=26); and 3) evaluate whether serum CTXI, a marker of type 1 collage degradation, is associated with MI in elephants (Asians: N=40; Africans: N=32). We used longitudinal serum and health data on ovarian cycling and non-cycling zoo African and Asian elephants with and without MI. Serum progestagen, leutinizing hormone, follicle stimulating hormone, and CTXI concentrations were measured. Cyclicity status was based on progestagen patterns. Zoo veterinarians classified the elephant with MI based on clinical exams and observations. In both Asian and African elephants, MI was significantly associated with cyclicity status (P=0.015, 0.032, respectively), but not independent of age (P=0.251, 0.091, respectively). There were no significant differences in any hormone concentration, during either the luteal or follicular phase, between elephants with or without MI (P>0.135). There were no significant differences in CTXI concentrations between age- and cycling-matched elephants with and without MI (P>0.957). Preliminary results do not provide compelling evidence that gonadotropin and sex hormones influence MI development in elephants.

